# Occupational therapy treatment of public safety personnel with work-related psychological injuries: analyzing Ontario worker’s compensation data from 2017–2021

**DOI:** 10.3389/fpsyt.2024.1377157

**Published:** 2025-01-06

**Authors:** Megan Edgelow, Agnieszka Fecica

**Affiliations:** School of Rehabilitation Therapy, Queen's University, Kingston, ON, Canada

**Keywords:** first responders, occupational health, occupational therapy (MeSH), posttraumatic stress disorder (PTSD), public safety personnel, return to work (RTW)

## Abstract

This study explored the usage of occupational therapy treatment with psychologically injured public safety personnel (PSP) from Ontario, Canada. We used a descriptive quantitative approach with summary data provided by the Workplace Safety and Insurance Board (WSIB) of Ontario documenting occupation therapy (OT) treatment of psychologically injured PSP who had an approved WSIB Mental Stress Injury Program (MSIP) claim between 2017 and 2021. Variables examined included demographics, career type, injury type, and return to work (RTW) outcomes. Chi-square Tests of Independence were used to compare differences between PSP who received OT treatment and those who did not. Analysis revealed that in the total cohort of 6674 approved PSP MSIP claims, 15% (n = 991) of PSP received OT treatment. Communicators (21%) and correctional workers (17%) were most likely to receive OT treatment while paramedics (13%) were less likely. PSP claimants who received OT treatment were more likely to have a cumulative event injury (71%) compared to the rest of the cohort (55%) and were more likely to not have started a RTW process (62%) compared to the rest of the cohort (43%). PSP who received OT treatment had more days away from work on average than those who did not (913 days vs. 384 days). This data reveals that PSP with cumulative injuries and higher lengths of time away from work more frequently received OT treatment as part of their WSIB MSIP claim; it is possible that this higher degree of claim complexity influenced their RTW outcomes. Worker’s compensation organizations should consider their health care decision-making processes to foster prompt access to treatment and proactive RTW pathways.

## 1 Introduction

Canadian “Public Safety Personnel” (PSP), including first responders, work to ensure public safety and include professionals such as communicators, correctional workers, firefighters, paramedics, and police officers (1). In their work, PSP are often exposed to potentially psychologically traumatic events (PPTE) known to contribute to the development of stress-related mental health conditions, including posttraumatic stress disorder (PTSD), depression, and anxiety disorders (2, 3). These conditions can impact mood, sleep, executive function, relationships, and the ability to work (4–7). A recent Canadian study assessed a large sample of PSP, with approximately two in five participants screening positive for one or more mental health disorders, primarily major depressive disorder (26.4%) and PTSD (23.2%) (2).

In Ontario, Canada, PSP with work-related psychological injuries may be eligible for support through the Workplace Safety and Insurance Board (WSIB) of Ontario – Mental Stress Injury Program (MSIP). This program offers a variety of benefits for injured workers including health care coverage, loss of earnings benefits, and return to work (RTW) support services. For many PSP organizations and affected communities, MSIP claims represent substantial costs, including wage replacement for employees on leave, backfilling positions, and healthcare related costs (8). The number of MSIP claims made by PSP workers has increased in Ontario in recent years; WSIB reported an increase in this type of claim from 0.3% in 2002 to 3% in 2020 (9), and a 2023 study documented an annual claim increase from 1,050 in 2017 to 1,420 in 2021 (10). Compounding these costs is the potentially lengthy nature of these claims (11), with half of the WSIB MSIP claims made between 2016 and 2020 lasting longer than 2 years (12).

A recent study of Ontario first responders showed that those with psychological injuries returned to work at a slower pace than those with musculoskeletal injuries and that claim and medical lag time delayed RTW (13). Further, a synthesis of systematic reviews has identified common factors that affect RTW outcomes after injury/illness; factors associated with positive RTW outcomes are lower injury/illness severity, RTW coordination, and multidisciplinary interventions that include the workplace and stakeholders (14). Common factors associated with negative RTW outcomes are older age, being female, and previous sick leave and unemployment (14).

Occupational therapists (OTs) are one of the health professions involved in delivering multidisciplinary interventions to support RTW after injury/illness (14, 15). In Ontario, OTs are increasingly involved in addressing the needs of PSP who have made a WSIB MSIP claim, and research in this area is limited but developing. A recent survey-based study revealed that Ontario PSP with MSIP claims were most frequently treated by general practitioners (44%), OTs (60%), and psychologists (61%) (15). This study also provided insight into the practices of OTs with this population, showing that PSP valued OT’s community based and practical approaches to RTW (13). A subsequent survey-based study of the OTs providing these RTW services in Ontario showed that this is a growing practice area and that more evidence is needed to describe the current state of practice with PSP (16).

Given the current lack of data on the usage of OT treatment with psychologically injured PSP, and the reported growing use of OT treatment with this population in Ontario, the goal of the current study was to investigate the usage of OT treatment in approved WSIB MSIP claims for PSP in Ontario, using available data from between 2017 and 2021. Specifically, this study sought to answer the following questions: What are the characteristics of PSP who received OT treatment as part of their MSIP claim? How do they compare to those who did not receive OT treatment? How do the RTW outcomes for these two groups compare?

## 2 Methods

This descriptive quantitative study explored WSIB MSIP claimant data documenting OT involvement in the treatment of work-related psychological injuries among PSP between January 1, 2017, and December 31, 2021. The data was accessed through a data sharing agreement between WSIB and the first author and captured the first five years after a legislation change in Ontario in 2016 had expanded eligibility for PTSD claims for Ontario PSP (10). To protect the privacy of the study population, the terms of the data sharing agreement stipulated that summary data, and not individual claim level data, would be provided by the WSIB data analysis team. Continuous variables were provided as means and categorical variables were provided as counts. This study received ethical review and approval through the Queen’s University Health Sciences Research Ethics Board (HS-REB).

The data consisted of 6674 claims, 991 of which included OT treatment arranged and funded by WSIB. The claims that received OT treatment were identified by existing WSIB billing codes for community-based OT treatment sessions. From 2017-2021, WSIB-funded OT treatment was typically provided through these community-based OT services. Thus, it is likely that most OT treatment for PSP with approved MSIP claims were captured by these billing codes. The data summary shared by WSIB did not specify how or why claimants were referred for treatment, nor how many specific OT treatment sessions they received.

The claim data included three categories of variables: (1) claimant specific variables (PSP career category, age, sex, years of job experience), (2) claim specific variables (nature of injury, mean number of days off work), (3) RTW variables (RTW successful, RTW unsuccessful, or no RTW participation). The RTW variables were defined by WSIB as “RTW successful” (an injured worker has resumed employment successfully); “RTW unsuccessful” (an injured worker was unable to return to employment); “no RTW participation” (an injured worker was never assigned to a RTW program). Data on the type of job that PSP returned to (e.g., own job, own employer; new job, own employer; new job, new employer) was not provided by WSIB. All MSIP categories were included: chronic mental stress, traumatic mental stress, and PTSD. To protect the privacy and confidentiality of claimants, WSIB did not share summary data that consisted of 5 or fewer claimants. As a result, a small percentage of the data was missing in some smaller career categories (see the Results tables for further detail). To facilitate statistical analysis, smaller career categories (by-law officers and commissioned police, fire chiefs and officers, managers in social, community, and correctional services and probation officers) were merged into one of five larger career categories: communicators, correctional workers, firefighters, paramedics, and police officers. See Data Variable Table for details (**Appendix A, Supplementary Material**).

Analyses focused primarily on exploring claims that included OT treatment and how these claims differed from those that did not include OT treatment. Continuous variables were provided by WSIB as means, and these are reported in the Results section and in the Tables. Categorical variables were provided by WSIB as counts, and it was possible to analyze these variables further to understand if there were any associations between those who received OT treatment and those who did not. Chi-Square Tests of Independence, which analyze the independence or association of categorical variables, were conducted to compare the observed and expected frequencies of the categorical variables. Data analysis was carried out using the Social Science Statistics Chi-Square Test Calculator, which is suitable for analyzing summary data.

## 3 Results

### 3.1 Overview of claimant data

Of the 6674 PSP claims, 14.8% of claims (n = 991) included occupational therapy treatment. Data on the average age and years of experience for claimants was provided by WSIB for each career category (**Table 1**). Claimants who received OT treatment were on average older (43.25 years) than those who did not (41.5 years). Information regarding the biological sex of claimants was available for 6655 claims. Females made up 33.3% (n = 2213) of the total sample but 40.8% (n = 399) of the claimants who received OT treatment. WSIB categorized MSIP claim injuries as either “single event” injuries or “cumulative event” injuries, with single event injuries resulting from a one-time traumatic event, while cumulative events resulted from multiple traumatic events or substantial work-related stressors. Of the 6674 PSP claims analyzed, nature of injury data was available for 6457 claims (**Table 2**). Overall, there were more cumulative event injury claims in the sample (57.6%, n = 3719) with 18.8% (n = 699) of those involving OT treatment, while only 9.3% (n = 254) of the single event claims (n = 2738) involved OT treatment.

**Table 1:** Demographics.

Total (n = 6665)	Communicators (n = 475, 7.1%)	Corrections (n = 1441, 21.6%)	Firefighters (n = 622, 9.3%)	Paramedics (n = 1897, 28.5%)	Police (n = 2230, 33.5%)
OT (n = 982)*	21.0% (100)	16.9% (243)	15.1% (94)	12.7% (241)	13.6% (304)
No OT (n = 5683)	79.0% (375)	83.1% (1198)	84.9% (528)	87.3% (1656)	86.4% (1926)
Mean Age (years)					
Total	42.5	41.1	46.5	37.8	43.1
OT	44.4	42.9	46.8	40.4	44.3
No OT	42.0	40.5	46.5	37.5	42.9
Job Experience (years)					
Total	16.1	19.1	23.5	14.2	18.8
OT	18.3	21.6	28.8	17.7	20.3
No OT	15.4	18.6	22.6	13.7	18.6
Female**					
Total (n =2213, 33.3%)	80% (380)	34.6% (496)	7.9% (49)	39.5% (749)	24.2% (539)
OT	77% (77)	42.3% (101)	8.5% (8)	47.7% (115)	32.2% (98)
No OT	80.8% (303)	33.1% (395)	7.8% (41)	38.3% (634)	22.9% (441)
Male**					
Total (n= 4442, 66.7%)	20% (95)	65.4% (936)	92.1% (573)	60.5% (1148)	75.8% (1690)
OT	23.0% (23)	57.7% (138)	91.5% (86)	52.3% (126)	67.8% (206)
No OT	19.2% (72)	66.9% (798)	92.2% (487)	61.7% (1,022)	77.1% (1,484)

No OT, Not Referred to Occupational Therapy; OT, Referred to Occupational Therapy.

Percentages in rows total vertically.

*0.13 % (n = 9) of claims were missing data on OT treatment status.

**0.28% (n= 19) of claims were missing data on sex.

**Table 2:** Claims data.

Total (n = 6457)*	Communicators (n = 468, 7.2%)	Corrections (n = 1361, 21.1%)	Firefighters (n = 603, 9.3%)	Paramedics (n = 1858, 28.8%)	Police (n =2167, 33.6%)
Treatment Status					
OT (n = 953)	21.2% (99)	16.9% (230)	15.3% (92)	12.7% (236)	13.7% (296)
No OT (n = 5504)	78.8% (369)	83.1% (1131)	84.7% (511)	87.3% (1622)	86.3% (1871)
Nature of Claim					
Single Event Claims					
Total (n = 2738, 42.4%)	35.5% (166)	43.9% (597)	22.6% (136)	60.1% (1116)	33.3% (723)
OT (n = 254)	26.3% (26)	25.2% (58)	16.3% (15)	39.8% (94)	20.6% (61)
No OT (n = 2484)	37.9% (140)	47.7% (539)	23.7% (121)	63.0% (1022)	35.4% (662)
Cumulative Event Claims					
Total (n = 3719, 57.6%)	64.5% (302)	56.1% (764)	77.4% (467)	39.9% (742)	66.6% (1444)
OT (n = 699)	73.7% (73)	74.8% (172)	83.7% (77)	60.2% (142)	79.4% (235)
No OT (n = 3020)	62.1% (229)	52.3% (592)	76.3% (390)	37.0% (600)	64.6% (1209)
Mean Days Off Work					
Total	512.1	477.7	522.1	269.7	467.4
OT (n = 991)	902.5	936.7	1,180.3	778.6	922.6
No OT (n = 5686)	408.1	384.4	403.6	195.6	395.4

No OT, Not Referred to Occupational Therapy; OT, Referred to Occupational Therapy.

Percentages in rows total vertically.

* 3.25% (n =217) of claims were missing data regarding the nature of the claim.

Across all five PSP career categories, claims that received OT treatment also involved more mean days off work (mean = 913.2 days) than claims that did not include OT (mean = 384.4 days) (**Table 2**). WSIB reported 3 types of RTW outcomes: RTW successful, RTW unsuccessful, and no RTW participation, meaning that an injured worker was never assigned to a RTW program (see **Appendix A**). Over half the total claimants had been assigned to a RTW program (54.5%, n = 3636), while 45.5% (n = 3038) had yet to be assigned to a RTW program at the time of data collection (**Table 3**). Of the claims that involved OT treatment (n = 991), 38.3% (n = 380) had been assigned to a RTW program, while 61.7% (n = 611) had yet to be assigned to a RTW program. Overall, for those assigned to a RTW program (n = 3636), success rates were high with 96.7% (n = 3517) successful in RTW, while 3.3% (n = 119) did not RTW successfully.

**Table 3:** RTW outcomes.

(n = 6674)	Totals	Communicators (n=475, 7.1 %)	Corrections (n=1441, 21.6%)	Firefighters (n=626, 9.4%)	Paramedics (n=1897, 28.4%)	Police (n=2235, 33.4%)
**Treatment Status**	OT (n = 991)	21.0% (100)	16.9% (243)	15.7% (98)	12.7% (241)	13.8% (309)
	No OT (n = 5683)	79.0% (375)	83.1% (1198)	84.3% (528)	87.3% (1656)	86.2% (1926)
RTW Successful						
**Total**	52.7% (3517)	44.4% (211)	54.4% (784)	35.8% (224)	68.6% (1302)	44.6% (996)
**OT**	355	32.0% (32)	28.4% (69)	38.8% (38)	46.9% (113)	33.3% (103)
**No OT**	3162	47.7% (179)	59.7% (715)	35.2% (186)	71.2% (1189)	46.4% (893)
RTW Unsuccessful						
**Total**	1.8% (119)	1.9% (9)	1.7% (24)	1.9% (12)	1.4% (26)	2.1% (48)
**OT**	25	3.0% (3)	2.8% (7)	2.0% (2)	2.5% (6)	2.3% (7)
**No OT**	94	1.6% (6)	1.4% (17)	1.9% (10)	1.2% (20)	2.1% (41)
No RTW Participation						
**Total**	45.5% (3038)	53.7% (255)	43.9% (633)	62.3% (390)	30.0% (569)	53.3% (1191)
**OT**	611	65.0% (65)	68.7% (167)	59.2% (58)	50.6% (122)	64.4% (199)
**No OT**	2427	50.7% (190)	38.9% (466)	62.8% (332)	27.0% (447)	51.5% (992)

No OT, Not Referred to Occupational Therapy; OT, Referred to Occupational Therapy; RTW, Return to Work.

Percentages in rows total vertically.

#### 3.1.1 Summary by career category

Of the 6674 claims analyzed, 475 were from communicators (7.1%), 1441 were from correctional workers (21.6%), 626 were from firefighters (9.4%), 1897 were from paramedics (28.4%), and 2235 were from police (33.4%). 991 claims (14.8%) included occupational therapy treatment, and career information was available for 982 of these claims.

21% of communicators (n = 100) were referred to OT. Those who were referred to OT were 44.4 years old with 18.3 years of experience on average, 77% were female and 73.7% had cumulative event claims. The OT group had a mean of 902.5 days off from work; 32% RTW successfully, 3% did not RTW and 65% were not assigned to a RTW program. The 375 communicators who were not referred to OT were younger at 42 years of age and 15.4 years of experience on average, 80.8% were female and 62.1% had cumulative event claims. They had fewer mean days off work at 408.1 days; 47.7% successfully RTW, 1.6% did not RTW and 50.7% were not assigned to a RTW program. See **Tables 1**–**3** for further detail.

16.9% of correctional workers (n = 243) were referred to OT. Those who were referred to OT were 42.9 years old with 21.6 years of experience on average, 57.7% were male and 74.8% had cumulative event claims. The OT group had a mean of 936.7 days off from work; 28.4% RTW successfully, 2.8% did not RTW and 68.7% were not assigned to a RTW program. The 1198 correctional workers who were not referred to OT were younger at 40.5 years of age and 18.6 years of experience on average, 66.9% were male and 52.3% had cumulative event claims. They had fewer mean days off work at 384.4 days; 59.7% successfully RTW, 1.4% did not RTW and 38.9% were not assigned to a RTW program. See **Tables 1**–**3** for further detail.

15.1% of firefighters (n = 94) were referred to OT. Those who were referred to OT were 46.8 years old with 28.8 years of experience on average, 91.5% were male and 83.7% had cumulative event claims. The OT group had a mean of 1180.3 days off from work; 38.8% RTW successfully, 2.0% did not RTW and 59.2% were not assigned to a RTW program. The 528 firefighters who were not referred to OT were younger at 46.5 years of age and 22.6 years of experience on average, 92.2% were male and 76.3% had cumulative event claims. They had fewer mean days off work at 403.6 days; 35.2% successfully RTW, 1.9% did not RTW and 62.8% were not assigned to a RTW program. See **Tables 1**–**3** for further detail.

12.7% of paramedics (n = 241) were referred to OT. Those who were referred to OT were 40.4 years old with 17.7 years of experience on average, 52.3% were male and 60.2% had cumulative event claims. The OT group had a mean of 778.6 days off from work; 46.9% RTW successfully, 2.5% did not RTW and 50.6% were not assigned to a RTW program. The 1656 paramedics who were not referred to OT were younger at 37.5 years of age and 13.7 years of experience on average, 61.7% were male and 37% had cumulative event claims. They had fewer mean days off work at 195.6 days; 71.2% successfully RTW, 1.2% did not RTW and 27% were not assigned to a RTW program. See **Tables 1**–**3** for further detail.

13.8% of police (n = 309) were referred to OT. Those who were referred to OT were 44.3 years old with 20.3 years of experience on average, 67.8% were male and 79.4% had cumulative event claims. The OT group had a mean of 922.6 days off from work; 33.3% RTW successfully, 2.3% did not RTW and 53.3% were not assigned to a RTW program. The 1926 police who were not referred to OT were younger at 42.9 years of age and 18.6 years of experience on average, 77.1% were male and 64.6% had cumulative event claims. They had fewer mean days off work at 395.4 days; 46.4% successfully RTW, 2.1% did not RTW and 51.5% were not assigned to a RTW program. See **Tables 1**–**3** for further detail.

### 3.2 Chi square analysis of claimant data by treatment status

Chi-Square Tests of Independence were performed for all available categorical variables (career category, sex, injury type, RTW status) to understand if there were any associations in the sample between those who received OT treatment and those who did not.

A significant relationship was found between OT treatment status and PSP career category, X^2^(4, N = 6665) = 28.74, p <.001. Follow-up comparison tests revealed that there were proportionally more communicators whose claims included OT treatment (OT, 10.2% vs. No OT, 6.6%), X^2^(1, N = 6665) = 16.26, p <.001. There were also proportionally more correctional workers whose claims included OT treatment (OT, 24.7% vs. No OT, 21.7%), X^2^(1, N = 6665) = 6.64, p = .01. Conversely, there were proportionally fewer paramedics whose claims included OT treatment (OT, 24.5% vs. No OT, 29.1%), X^2^(1, N = 6665) = 8.7, p = .003. Further tests revealed that the proportion of claims made by police and firefighters did not differ significantly as a function of OT treatment status (all p >.05). A significant relationship was found between OT treatment status and sex, revealing that proportionally more females had claims that included OT treatment (OT, 40.8% vs. No OT, 32%) and conversely, proportionally fewer males received OT treatment (OT, 59.2% vs. No OT, 68%), X^2^(1, N = 6655) = 29.4 p <.001. See **Table 1** for claimant demographics.

Proportionally more cumulative claims included OT treatment (OT, 73.7% vs. No OT, 62.1%) compared to single events claims (OT, 26.3% vs. No OT, 37.9%), X^2^(1, N = 6457) = 113.57, p <.001. Additionally, cumulatively injured workers from all PSP career categories, except for firefighters, were proportionally more likely to receive OT treatment than those with single event injuries: communicators, X^2^(1, N = 468) = 4.65, p = .03 (cumulative, 15.6% vs. single, 5.5%); corrections, X^2^(1, N = 1361) = 39.1, p <.001 (cumulative, 12.6% vs. single, 4.3%); paramedics, X^2^(1, N = 1858) = 46.14, p <.001 (cumulative, 7.6% vs. single, 5.1%), and police, X^2^(1, N = 2167) = 25.01, p <.001 (cumulative, 10.8% vs. single, 2.8%). See **Table 2** for further detail. Proportionally fewer claimants whose claims included OT treatment had been assigned to a RTW program (OT, 38.3% vs. No OT, 57.3%) and conversely more claimants whose claims included OT treatment had not yet been assigned to a RTW program (OT, 61.7% vs. No OT, 42.7%), X^2^(1, N = 6674) = 122.17, p <.001. Further, fewer PSP whose claim included OT treatment successfully RTW (OT, 35.8% vs. No OT, 55.6%), X^2^(1, N = 3636) = 14.7, p <.001. See **Table 3** for RTW outcomes.

## 4 Discussion

This descriptive quantitative study explored WSIB Ontario claims data documenting OT treatment for psychologically injured PSP who had an approved MSIP claim between 2017 and 2021. This study sought to compare the MSIP claims of PSP who received OT treatment to those who did not, focusing on the demographic characteristics of claimants, claim complexity, and RTW outcomes. This study, centered on the worker’s compensation usage of occupational therapy treatment with psychologically injured PSP, is the first of its kind in Ontario, Canada, and beyond. Existing literature relevant to worker’s compensation, PSP, occupational therapy, and return to work is discussed in the following sections.

### 4.1 Claim characteristics and complexity

Of the 6674 PSP claims analyzed, 15% included occupational therapy treatment, and communicators had the highest proportion of claims involving OT treatment, followed by corrections, firefighters, police, and paramedics. A previous study of these WSIB MSIP claims found that when compared to their peers, paramedics had fewer cumulative injuries and were more likely to return to work than other PSP career groups (10). Since this study suggests that claims with higher complexity (as indicated by cumulative injuries and higher days off work) were sent to OT at proportionally higher rate, it is perhaps unsurprising that paramedics, the one PSP career group with more single event injury claims, received OT treatment less frequently. Paramedics were also the youngest group in the sample with the fewest years of experience on the job. Given that those who received OT treatment were on average older than the PSP who did not receive OT treatment, the lower rates of OT treatment observed among paramedics is in keeping with this finding.

This study also revealed that proportionally more female PSP than male received OT treatment. Given that communicators were the one PSP category where females outnumbered males, it is logical that more communicators received OT treatment. Previous studies found that women and female identifying PSP are more likely to be diagnosed with mental health conditions and to seek help; the higher rates of OT treatment observed among communicators in this sample may be reflective of these findings (17).

The study results suggest that claims that included OT treatment were more complex for a variety reasons. They were made by older PSP with more years of experience on the job, and proportionally more of these claimants were female. Additionally, claims that included OT treatment were more likely to be cumulative in nature. Claims that included OT treatment also involved on average more days off work than claims that did not include OT. These findings echo previous studies that found that exposure to PPTE increases with years of experience (17) and support the idea that more complex cases received OT treatment.

### 4.2 Return to work outcomes

PSP who received OT treatment as part of their WSIB MSIP claim had slightly lower rates of successful RTW and a higher likelihood of not having started a RTW process compared to those who did not receive OT treatment. The claim duration was also much longer for those who received OT treatment (approximately 2.5 years) compared to those who did not (approximately 1 year). A previous study of this cohort also found that PSP waited on average 1.5 years to be referred to OT after their WSIB MSIP claim was approved (18). In addition, those who received OT treatment had indicators of higher claim complexity, including being older on average, having a higher proportion of cumulative injuries, and having longer claim durations. Previous research on RTW factors for a variety of populations, including those with mental health conditions, indicates that key risk factors to unsuccessful RTW include age, female sex, comorbidities, and severity of injury (14, 19). Additionally, a previous study of Ontario PSP showed that most PSP did not RTW after posttraumatic stress injury rehabilitation, although there were improved outcomes with earlier intervention (13).

There is no publicly available information that explains decision-making processes within WSIB related to referrals to healthcare professionals for claimants. Considering the outcomes observed in this study, and the broader literature on RTW factors, it appears that since WSIB is referring older PSP with higher levels of injury severity to OT, and that these referrals often come late in the claim duration, these PSP would naturally be less likely to RTW. Additionally, there may be contextual factors for different PSP career groups that influence the RTW outcomes; for example, firefighters were the oldest group at 47 years of age on average, and those closer to retirement age may have had less reason to attempt a RTW process.

### 4.3 Key takeaways from this study

Occupational therapy is often used with populations who have complex health conditions, including both physical and mental health injuries, illnesses, and related disabilities; in this study it is not surprising that the cohort who received OT treatment had indicators of higher injury severity and lower RTW rates. However, in this cohort, delays in access to OT, which were 1.5 years on average (18), may have had an outsize impact on the outcomes for this group. Gross et al. (20) found that Ontario PSP who received earlier rehabilitation for their posttraumatic stress injuries had improved RTW outcomes, and a recent study from WSIB showed that earlier access to mental health interventions for traumatic brain injury (TBI) claimants predicted earlier RTW outcomes (21). A Canadian study of first responders receiving disability management for workplace injuries found that predictors of RTW included injury type, with mental health claims having longer duration, and claim and medical time lag delaying RTW (13). Additionally, another recent study of Ontario police officers revealed that timeliness was an important factor in the RTW journeys of these PSP (22).

With lengthy worker’s compensation claim durations comes rising economic costs, further making the case for earlier access to rehabilitation services like OT. A recent economic analysis of the impact of OT on RTW for people with mental health conditions demonstrated positive RTW outcomes along with cost savings for several RTW models (23). Additionally, this analysis showed that adding OT to a program for depression improved outcomes and had an economic benefit.

This study’s findings are a first step in understanding how OT treatment is used by WSIB Ontario with PSP MSIP claims. As PSP are a group with lower RTW rates compared to other WSIB claim types (10), regardless of OT treatment status, representing significant human and financial costs, this issue needs further study. Future research should investigate claim level data to understand the risk factors that are driving claim length and lack of RTW readiness. Additionally, data that expand on how WSIB is using community OT treatment and other factors that may influence RTW outcomes (e.g. specific injury, number of OT treatment sessions, other healthcare professionals and their treatment counts, medications, career stage) should be explored. This future research can help workers compensation organizations update their return to work processes and utilize occupational therapy, and other health care professionals, optimally.

### 4.4 Study limitations

In this study, the data provided by WSIB was in summary format, limiting potential statistical tests and the research team’s ability to explore claim-level variables. Additional demographic variables like socioeconomic status, race, and marital status were not available and their impact on outcomes could not be analyzed. Additionally, standard deviations and ranges were not provided by WSIB for continuous variables and statistical analyses on means could not be conducted. Since the data set was from 2017-2021, recent changes to this data are unknown. It is possible that changes in RTW status or OT referral status occurred for some PSP after 2021, particularly for those with claims that were made later in the five-year period. The results draw on data from Ontario, Canada, which may limit generalizability to other contexts and worker’s compensation systems.

## 5 Conclusion

This study is the first to describe the usage of occupational therapy treatment for psychologically injured PSP in Ontario. Using summary data, it compared WSIB MSIP claims for Ontario PSP who received OT treatment to those who did not for the years 2017 to 2021. PSP who received OT treatment were more likely to be female, to be older and have more years of experience, and to have cumulative injuries. The cohort who received OT treatment were more likely to not have started a RTW process and had longer overall claim durations. Past research on this cohort also shows that this group waited on average 1.5 years into their claim duration to begin OT treatment. RTW research shows that earlier access to rehabilitation for PSP and other populations is associated with better RTW outcomes. Since the OT treatment referral decision-making processes and pathways within WSIB are not publicly known, it would be beneficial for WSIB to review its processes and consider streamlining access to OT, particularly considering its demonstrated economic benefits with clients with mental health conditions. Researchers could seek a more detailed data set in order to consider long term return to work trajectories, as well as modelling the impact of variables on return to work status and OT referral status. Further research is needed to understand the reasons for the current usage of OT treatment within WSIB, and other claim-level factors that may be influencing RTW outcomes for those referred for OT treatment, to make more comprehensive recommendations for the usage of OT treatment with psychologically injured PSP.

## Data Availability

The original data set used in this study is owned by the Workplace Safety Insurance Board of Ontario and is not publicly available due to privacy guidelines. Requests to access the datasets should be directed to edgelowm@queensu.ca.
