# Supplementary material for: Occupational therapy treatment of public safety personnel with work-related psychological injuries: analyzing Ontario worker’s compensation data from 2017–2021
Source: Front Psychiatry. 2025 Jan 6;15:1377157. doi: 10.3389/fpsyt.2024.1377157 (PMC11743646; doi:10.3389/fpsyt.2024.1377157)
Supplement: Supplementary file 1 [file DataSheet1.pdf]

Appendix A: Data Variables

Variable Group	Variables Name	Variable Categories	Notes
**Claimant Variables**			
	Age	Age at Accident	Age of the worker in years on the date the injury/illness occurred
	Job Experience	Years of Experience	Number of years between the employment date and the date of injury/illness
	Sex	• Male<br>• Female	
	PSP Occupation	• Communicators<br>○ 1475 – Dispatchers and Radio Operators<br>• Corrections<br>○ 6462 – Correctional Services Officers<br>○ 4155 – Probation and Parole Officers and Related Occupations<br>○ 0314 – Managers in Social, Community and Correctional Services<br>• Firefighters<br>○ 6262 – Fire-fighters<br>○ 0642 – Fire Chiefs and Senior Fire-fighting Officers<br>• Paramedics<br>○ 3234 – Ambulance Attendants and Other Paramedical Occupations<br>• Police<br>○ 6261 – Police Officers (except Commissioned)<br>○ 0641 – Commissioned Police Officers<br>○ 6463 – By-law Enforcement and Other Regulatory Officers, n.e.c.	Based on the NOC Codes (NOC 2006 Version 1.0).
**Claim Variables**			
	Nature of Injury	• Single Event<br>• Cumulative Events	Categorizes a claim by the nature of injury into 2 categories of 1) Single Event 2) Cumulative Events
	Days Off Work	Lost Time from Work	Time away from work due to injury/illness in days over the duration of a claim
**RTW Variables**			
	RTW Outcomes	• RTW Successful<br>• RTW Unsuccessful<br>• No RTW Participation	"RTW successful"(an injured worker has resumed employment successfully); "RTW unsuccessful" (an injured worker was unable to return to employment); "no RTW participation" (an injured worker was never assigned to a RTW program)
